# Impact of opioid-free anesthetic on postoperative complication following gynecological surgery: a meta-analysis

**DOI:** 10.1590/1980-220X-REEUSP-2025-0218en

**Published:** 2025-12-01

**Authors:** Lihua Yang, Fengming Tu

**Affiliations:** 1 Huzhou Maternity & Child Health Care Hospital, Department of Emergency, Huzhou, China.; 2 Huzhou Maternity & Child Health Care Hospital, Department of Obstetrics, Huzhou, China.

**Keywords:** Gynecologic Surgical Procedures, Analgesics, Non-Narcotic, Postoperative Nausea and Vomiting, Postoperative Complications, Pain, Postoperative, Procedimentos Cirúrgicos em Ginecologia, Analgésicos não Narcóticos, Náusea e Vômito Pós-Operatórios, Complicações Pós-Operatórias, Dor Pós-Operatória

## Abstract

A meta-analysis study was performed to scrutinize how opioid free anesthesia impact on the postoperative complication following gynecological surgery (GS). The 10 selected studies such as 852 females who underwent GS at the beginning of the study. Odds ratios (ORs) and mean differences (MDs) and 95% confidence intervals (CIs) were used to analyzed the effects of O-FA as compared to control treatment (opioid based anesthetic) on GS dichotomous or continuous methods using either a fixed or random effects model. The females undergoing GS, O-FA was associated with significantly lower postoperative nausea and vomiting (OR, 0.33; 95% CI, 0.24 – 0.46, p < 0.001), lower rescue antiemetic (OR, 0.32; 95% CI, 0.13 – 0.78, p = 0.01) and higher Quality of Recovery-40 (MD, 4.69; 95% CI, 2.48 – 6.90, p < 0.001) compared to control treatment. However, no significant difference was observed between in extubation (MD, – 0.61; 95% CI, –1.45 – 0.22, p = 0.15), postoperative pain score (MD, 0.49; 95% CI, –0.64–1.62, p = 0.39), and analgesic use (OR, 1.77; 95% CI, 0.57–5.50, p = 0.33) in females with GS. In females undergoing GS, O-FA resulted in significantly lower postoperative nausea and vomiting, reduced need for rescue antiemetic, and higher Quality of Recovery-40 compared to opioid-based anesthesia.

## RESUMO

Foi realizado um estudo de meta-análise para examinar o impacto da anestesia sem opióides nas complicações pós-operatórias após cirurgia ginecológica (CG). Os 10 estudos selecionados incluíram 852 mulheres submetidas a CG no início do estudo. Foram utilizadas razões de probabilidades (OR), diferenças médias (DM) e intervalos de confiança (IC) de 95% para analisar os efeitos do O-FA em comparação com o tratamento controlo (anestésico à base de opióides) na CG por métodos dicotómicos ou contínuos, utilizando um modelo de efeitos fixos ou aleatórios. Nas mulheres submetidas a GS, o O-FA foi associado a náuseas e vómitos pós-operatórios significativamente mais baixos (OR, 0,33; IC 95%, 0,24 – 0,46, p < 0,001), menor antiemético de resgate (OR, 0,32; IC 95%, 0,13 – 0,78, p = 0,01) e maior Qualidade de Recuperação-40 (MD, 4,69; IC 95%, 2,48 – 6,90, p<0,001) em comparação com o tratamento de controlo. No entanto, não se verificou diferença significativa entre a extubação (DM, −0,61; IC 95%, −1,45 – 0,22, p = 0,15), o score de dor pós-operatória (DM, 0,49; IC 95%, −0,64 – 1,62, p = 0,39) e o uso de analgésicos (OR, 1,77; IC 95%, 0,57 – 5,50, p = 0,33) nas mulheres com GS. Nas mulheres submetidas a GS, a O-FA resultou em náuseas e vómitos pós-operatórios significativamente menores, menor necessidade de antiemético de resgate e maior Quality of Recovery-40 em comparação com a anestesia com opióides.

## INTRODUCTION

Opioids play a significant role in general anesthesia via providing the strong intraoperative anti-nociceptive effects and analgesia, while also helping to maintain stable vital signs during surgical procedures^1,2)^. Therefore, inappropriate opioid use can result in various adverse effects viz., postoperative nausea and vomiting (P-ONV), respiratory depression, inflammatory responses, and hyperalgesia, which extend the duration of postoperative hospitalization and elevate medical expenses^3)^. Moreover, postoperative opioid use may lead to opioid addiction and abuse^4)^. The prevalence of P-ONV in patients undergoing gynecological surgery (GS) with opioid administration ranges from 50% to 80%, and these patients often experience bradycardia and postoperative hypoxemia due to residual opioid effects^5,6)^. The growing emphasis on increased recovery after surgery has led to the enhancing adoption of O-FA across various surgical procedures^7)^. O-FA is a multimodal anesthesia approach that combines various pharmacological agents, including sedatives, N-methyl-D-aspartate receptor antagonists, local anesthetics, anti-inflammatory medications, and α^2^ receptor agonists to optimize intraoperative analgesia and minimize opioid use during the perioperative phase. Elkassabany and Mariano^8)^ describe O-FA as a perioperative strategy extending from admission to discharge, focusing on non-opioid technique for anesthesia and analgesia, along with opioids reserved only for pain unmanageable by alternative techniques. Forget^9)^ propose that O-FA represents a combination of multiple opioid sparing strategies to achieve complete avoidance of opioids. Mulier and Dekock^10)^ distinguish O-FA from opioid-free analgesia, emphasizing that O-FA involves no opioid administration prior to or during surgery until the patient regains consciousness. It can mitigate the risk of prevalent opioid-related side effects, comprising postoperative respiratory depression and P-ONV, while also diminishing the likelihood of patient reliance and addiction to opioids^11)^. O-FA has been successfully implemented in bariatric and thoracic surgeries with optimal outcomes^12,13)^. This meta-analysis aimed to compare and summarize postoperative outcome P-ONV, antiemetic use, pain scores, analgesic consumption, extubation time, and Quality of Recovery-40 (QoR-40) scores between O-FA and opioid-based anesthesia in gynecological surgery (GS).

## OBJECTIVES

The objective of study was to analyze the meta-analysis to scrutinize the effect of O-FA on postoperative complication following GS.

## METHODS

### Eligibility Criteria

To provide an overview of studies scrutinize the effect of O-FA on postoperative complication following GS^14)^.

### Information Sources

**Figure 1:**
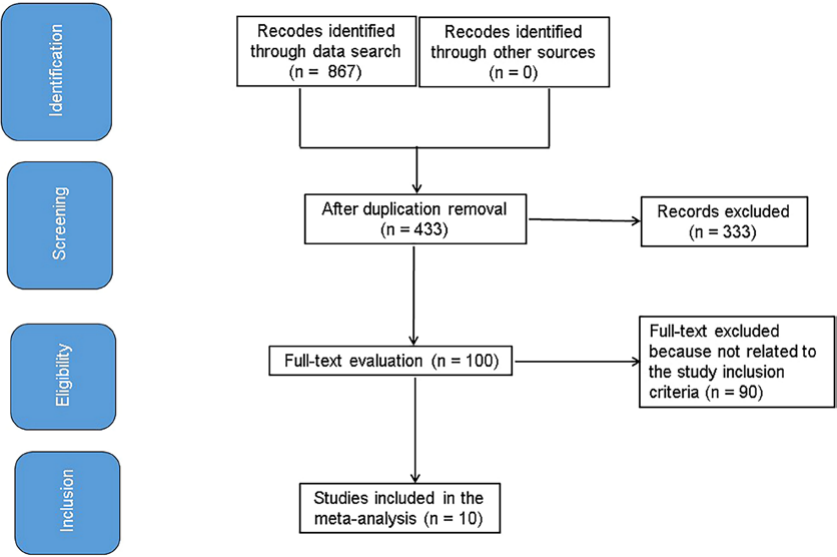
Procedure flowchart for the research. Zhejiang, China.

The full investigation is presented in Figure 1. The studies were included if they met the following inclusion criteria were met^15,16)^:

1. Studies were observational, prospective, retrospective or randomized controlled trials (RCTs). 1. Studies were observational, prospective, retrospective or randomized controlled trials (RCTs).
2. The participants were females who underwent for examination were females who had GS.
3. Postoperative pain was included into the study.
4. The study compared the effect of O-FA on postoperative complication following GS.

Studies were excluded the outcome of the O-FA only compared to control in GS, includes only the control treatment group or lacked statistically significance comparisions^17,18)^.

### Search Strategy

The PICOS perspective was applied to identify the search protocol procedure, which was characterized as follows: the P “population” such as females undergoing GS; O-FA was the I “intervention” was opioid free anesthesia (O-FA); C “comparison” was O-FA; O “outcome” included postoperative complications; and the S “study design” had no restrictions^19)^. Using a set of keywords and search terms (Chart 1), we conducted a comprehensive search of the Cochrane Library, Google Scholar, Embase, PubMed, and OVID databases through February 2025^20,21)^. To avoid including studies that were not relevant to the effect of O-FA on postoperative complication following GS, we also removed the duplicate records. The remaining articles were compiled into an EndNote database, and their titles, abstracts were screened for evaluation^22,23)^.

**Chart 1:** Database Search Strategy for inclusion of examinations Zhejiang, China

Database	Search strategy
**Google Scholar**	#1 “gynecological surgery” OR “opioid-free anesthetic” #2 “postoperative nausea and vomiting” OR “postoperative complication” OR “postoperative pain” #3 #1 AND #2
**Embase**	#1 ‘gynecological surgery’ /exp OR ‘opioid-free anesthetic’ #2 ‘postoperative nausea and vomiting’/exp OR ‘postoperative complication’/exp OR ‘postoperative pain’ #3 #1 AND #2
**Cochrane library**	#1 (gynecological surgery):ti,ab,kw OR (opioid-free anesthetic):ti,ab,kw (Word variations have been searched) #2 (postoperative nausea and vomiting):ti,ab,kw OR (postoperative complication):ti,ab,kw OR (postoperative pain):ti,ab,kw (Word variations have been searched) #3 #1 AND #2
**Pubmed**	#1 “gynecological surgery”[MeSH] OR “opioid-free anesthetic”[All Fields] #2 “postoperative nausea and vomiting”[MeSH Terms] OR “postoperative complication”[MeSH] OR “postoperative pain”[All Fields] #3 #1 AND #2
**OVID**	#1 “gynecological surgery”[All Fields] OR “opioid-free anesthetic” [All Fields] #2 “postoperative nausea and vomiting”[ All fields] OR “postoperative complication”[All Fields] OR “postoperative pain” [All Fields] #3 #1 AND #2

### Selection Process

The subsequent process was organized and analyzed according to the epidermiological guidelines and scrutinized using the meta-analysis methods^24,25)^.

### Data Collection Process

In this study, we extract the data such as author’s name, research data, research year, country or location, population type, categories, quantitative and qualitative estimating methodologies, data sources, consequence estimation, medical and treatment physiognomies, and statistical analysis were some of criteria used to collect data^26)^.

### Data Items

When a study reported the diverse values, data were extracted based on the evaluating the effect of O-FA on the postoperative complication following GS.

### Research Risk of Bias Assessment

The risk of bias and methodological quality of the selected investigation were independently scrutinized by two authors. Each study’s methodology was objectively reviewed to ensure the accuracy and reliability.

### Effect Measures

Sensitivity analysis was restricted to studies evaluating the effect of O-FA on postoperative complication following GS. A subclass analysis was carried out to compare the relationship between O-FA and control (opioid anesthetic) in diverse patients’ variables in GS patients’ sensitivity.

### Synthesis Methods

The 95% CI, mean difference (MD), and odds ratio (OR) were calculated for continuous and dichotomous data approach using either fixed-effect or random effect models. Heterogeneity was calculating the I^2^ statistic, with value of 0% to 100% was employed. No, low, moderate, and high heterogeneity were seen at 0%, 25%, 50%, and 75% of the data, respectively^27)^. Additional analyses of the studies with similar characteristics were carried out to ensure the appropriate model selection. If I^2^ was less than 50%, the fixed-effect was chosen; if not, the random effect was applied^27)^. By dividing the initial estimation into the previously designated consequence groups, a subclass analysis was carried out. The analysis used a p-value of less than 0.05 to determine if changes between subcategories were statistically significant.

### Reporting Bias Assessment

The Egger regression test and funnel plots, plotting the logarithm of ORs or MDs against their standard errors, were used as quantitative and qualitative techniques to assess the publication bias. A p ≥ 0.05 indicated the presence of inquiry bias^28)^.

### Certainty Assessment

Two-tailed tests were applied for all p-value. Reviewer Manager Version 5.3 (The Nordic Cochrane Centre, the Cochrane Collaboration, Copenhagen, Denmark) was used for statistical analyses and graphical representations.

## RESULTS

**Figure 2:**
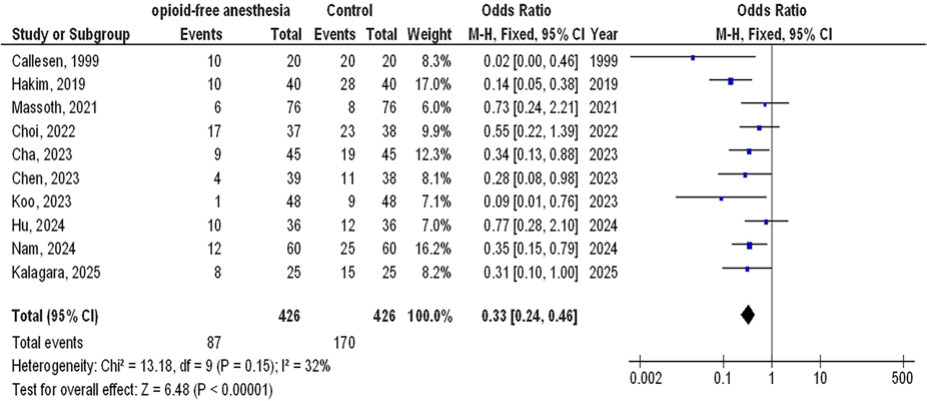
The opioid-free anesthetic compared to control treatment’s forest plot influence on postoperative nausea and vomiting in participants with gynecological surgery Zhejiang, China.

**Figure 3:**
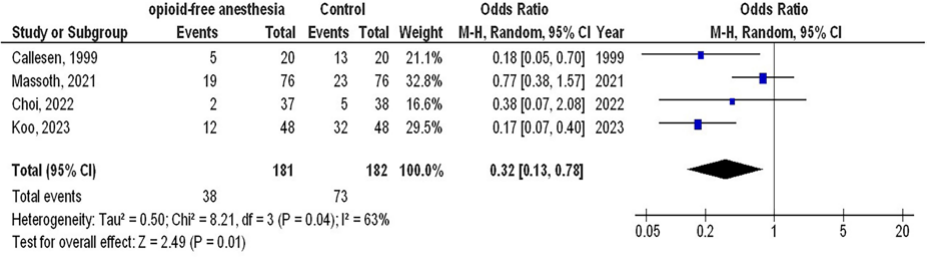
The opioid-free anesthetic compared to control treatment’s forest plot influence on rescue antiemetic in participants with gynecological surgery Zhejiang, China.

**Figure 4:**
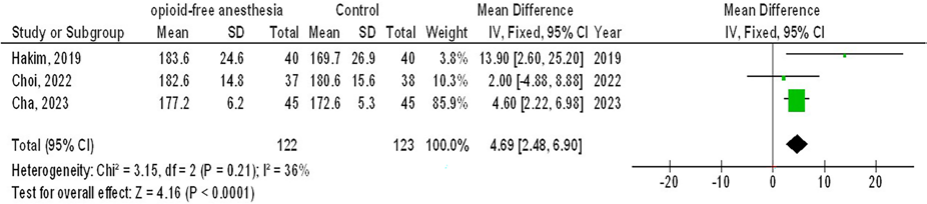
The opioid-free anesthetic compared to control treatment’s forest plot influence on QoR-40 in participants with gynecological surgery Zhejiang, China.

A total of 10 publications that met the inclusion criteria and were published between 1999 and 2025 were selected for the study from 867 related studies^(29–38)^. All selected investigation were randomized controlled trials. The results of these studies are showed in Table 1. In total, 852 females with GS were included at the start of the investigations. The sample size of the selected studies ranged from 40 to 152 patients. As illustrated in Figures 2–4, in females with GS, O-FA had significantly lower P-ONV (OR, 0.33; 95% CI,0.24 – 0.46, p < 0.001) with low heterogeneity (I^2^ = 32%), lower rescue antiemetic (OR, 0.32; 95% CI, 0.13–0.78, p = 0.01) with moderate heterogeneity (I^2^ = 63%), and higher QoR-40 (MD, 4.69; 95% CI, 2.48 – 6.90, p < 0.001) with low heterogeneity (I^2^ = 36%) compared to control treatment (opioid anesthetic).

**Table 1:** Qualities of the chosen studies for the meta-analysis – Zhejiang, China.

Study	Country	Total	Opioid-free anesthesia	Control
Callesen, 1999^29)^	Denmark	40	20	20
Hakim, 2019^30)^	Egypt	80	40	40
Massoth, 2021^31)^	Germany	152	76	76
Choi, 2022^32)^	Korea	75	37	38
Cha, 2023^33)^	China	90	45	45
Chen, 2023^34)^	China	77	39	38
Koo, 2023^35)^	Korea	96	48	48
Hu, 2024^36)^	China	72	36	36
Nam, 2024^37)^	Korea	120	60	60
Kalagara, 2025^38)^	India	50	25	25
	Total	852	426	426

**Figure 5:**
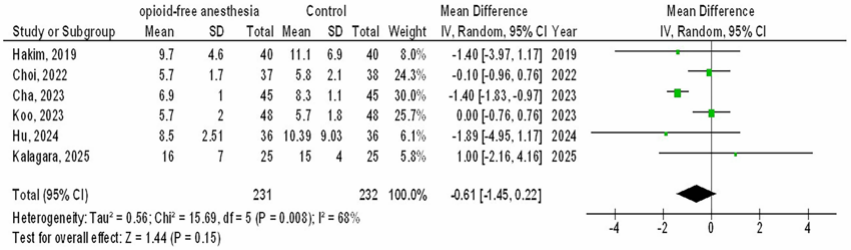
The opioid-free anesthetic compared to control treatment’s forest plot influence on time of extubation in participants with gynecological surgery Zhejiang, China.

**Figure 6:**
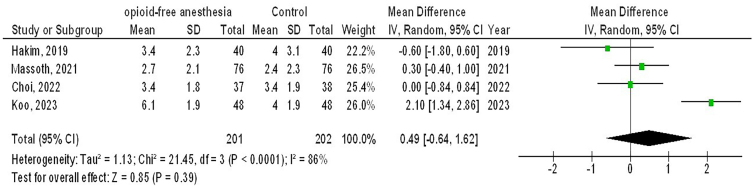
The opioid-free anesthetic compared to control treatment’s forest plot influence on postoperative pain score in participants with gynecological surgery Zhejiang, China.

**Figure 7:**
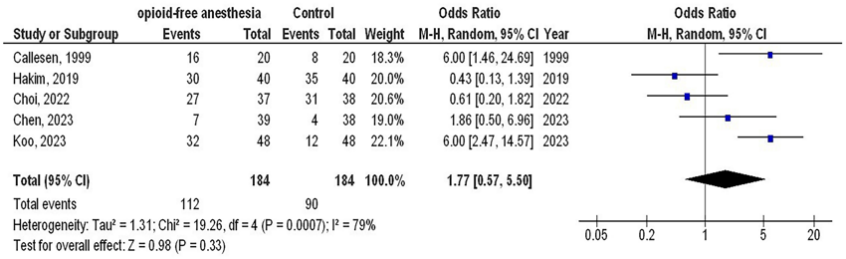
The opioid-free anesthetic compared to control treatment’s forest plot influence on analgesic use in participants with gynecological surgery Zhejiang, China.

However, no significant differences were observed between O-FA and control treatment in time of extubation (MD, −0.61; 95% CI, −1.45 – 0.22, p = 0.15) with moderate heterogeneity (I^2^ = 68%), postoperative pain score (MD, 0.49; 95% CI, −0.64 – 1.62, p = 0.39) with high heterogeneity (I2 = 86%), and analgesic use (OR, 1.77; 95% CI, 0.57 – 5.50, p = 0.33) with high heterogeneity (I2 = 79%) in females with GS as shown in Figures 5–7.

The use of stratified models to assess the effects of specific factors were not feasible sur to insufficient data, such as gender, ethnicity, and age, on comparison consequences. No evidence of publication bias was found using the visual clarification of funnel plot and quantitative Egger regression test (p = 0.88, 0.89, 0.89, 0.91, 0.86, and 0.91, respectively). However, most of the included RCTs exhibited suboptimal methodology quality, although no evidence of the selecting reporting bias was observed.

## DISCUSSIONS

Further research is warranted to validate these findings, as several comparisons were based on a limited number of studies with small sample sizes, which may influence the reliability of the results.

The meta-analysis such as 852 females with GS^(29–38)^. In the females, O-FA was related with significantly lower P-ONV, reduced need for the rescue antiemetic, and higher QoR-40 scores compared to the control treatment (opioid anesthetic). However, no significant difference was observed differences were observed in the time to extubation, postoperative pain score, or analgesic use in females with GS. Further research is needed to confirm these findings and caution should be exercised when the interpreting the results, as various comparisons were based on a limited number of studies, many of which had small sample sizes. This may affect the overall reliability and significance of the reviewed assessments.

Despite numerous previous research examining the postoperative results of O-FA and opioid-based anesthesia, there remains a lack of systematic reviews and meta-analyses focusing on a specific surgical procedure. Two meta-analyses reported that O-FA reduced postoperative pain in patients^39,40)^, and one indicated a shorter extubation time^40)^, which contrasts with our findings. O-FA substitutes opioids with alternative medications or anesthetic methods during anesthesia, hence reducing perioperative opioid use, which may decrease the occurrence of P-ONV, aligning with the findings of this meta-analysis. Callesen et al. employed combined spinal-epidural anesthesia in the O-FA group^29)^, and regional anesthesia may present a distinct risk of P-ONV in comparison to general anesthesia, thereby influencing the data’ accuracy^41)^. P-ONV remains to significant clinical challenge. Female sex is an intendent risk factors for P-ONV, with a markedly higher prevalence observed in GSs compared to other types of surgeries^42)^. Apfel et al. reported that the incidence of P-ONV can reach upto 80% in GSs undergoing opioid-based anesthesia^5,43)^. Although opioids are commonly used as perioperative analgesics due to their potent pain relieving effects, they are also related with side effects, including nausea and vomiting^44)^. Mechanistically, opioids can induces P-ONV via direct stimulation of receptors in the chemoreceptor trigger zone of the brainstem^45)^. Substantial evidence further shows that both the prevalence and severity of P-ONV are dose-dependent on perioperative opioids^46)^.

Among the included investigations, four incorporated dexmedetomidine as part of O-FA. Previous research indicates that dexmedetomidine, an α^2^ receptor agonist, may diminish the incidence of P-ONV by modulating the release of 5-hydroxytryptamine and dopamine^47)^. Despite the variable combinations of anesthetic agents. Despite the varying combinations of anesthetic agents across studies, our findings suggest that O-FA provides a benefit in reducing P-ONV during gynecological procedures. The effect of O-FA on postoperative pain remains inconclusive; this meta-analysis found no statistically significant differences in postoperative pain scores or analgesic requirements between the O-FA and control groups. These findings support the use of the multimodel analgesia to reduce opioid consumption and achieve adequate pain relief. However, variations in postoperative analgesic protocols and multimodal analgesia regimens across studies limits the reliability of this conclusion, highlighting the need for further clinical trials to better define the relationship between O-FA and postoperative pain. Additionally, no significant difference in extubation time was observed between two groups. While opioid use is generally expected to prolong extubation due to increased sedation, this finding contradicts conventional assumptions.

No difference in extubation time was observed between the two groups. Although opioid use is typically excepted to increase sedation and prolong extubation time; this finding contradicts conventional assumptions and may be attributed to the limited sample size, which reduces its reliability. The meta-analysis also showed that O-DA enhanced the recovery quality. The included investigation assessed recovery quality. The included investigation scrutinized recovery using the QoR-40 scale, in which P-ONA is a significant element of QoR-40 scale; therefore, the reduced occurrence of P-ONV may enhance postoperative recovery quality. Nevertheless, the restricted number of included studies and significant variability restrict the confidence in secondary outcomes.

## LIMITATIONS

The primary limitation of this review is that most included investigations lacked data on lumbar stability, degree of slippage and patients primary symptom, preventing further stratified analysis. Additionally, insufficient information was available to scrutinize the potential effect of age, sex or race on outcome. This restriction prevented us from performing additional stratified analysis. Furthermore, we lacked sufficient data to assess the potential influence of age, sex, and race on outcomes. The inclusion of incomplete or inaccurate data from previous investigation may have introduced bias. However, unreported investigation and missing information such as participants’ age, gender, race, and nutrition were probably biassed. Incomplete data and unreported research may result in values that are inadvertently skewed.

## CONCLUSIONS

In females with undergoing gynecological surgery, O-FA was associated with significantly lower P-ONV, reduced need for rescue antiemetic, and higher QoR-40 score compared to control treatment (opioid anesthetic). However, no significant differences were observed in extubation time, postoperative pain score and analgesic use. Further research is required to validate these finding, as various comparisons were based on a limited number of investigations, various with small sample sizes, which may affect the reliability and significance of the results.

## Data Availability

The datasets analyzed during the current study are available from the corresponding author upon reasonable request.
