# Supplementary material for: Impact of opioid-free anesthetic on postoperative complication following gynecological surgery: a meta-analysis
Source: Rev Esc Enferm USP. 2025 Dec 1;59:e20250218. doi: 10.1590/1980-220X-REEUSP-2025-0218en (PMC12704943; doi:10.1590/1980-220X-REEUSP-2025-0218en)
Supplement: Translation

# Translation: Impacto de la anestesia sin opioides en las complicaciones posoperatorias tras una cirugía ginecológica: un metaanálisis

**DOI:** 10.1590/1980-220X-REEUSP-2025-0218es

**Authors:** Lihua Yang, Fengming Tu

**Affiliations:** 1 Huzhou Maternity & Child Health Care Hospital, Department of Emergency, Huzhou, China.; 2 Huzhou Maternity & Child Health Care Hospital, Department of Obstetrics, Huzhou, China.

**Keywords:** Procedimientos Quirúrgicos Ginecológicos, Analgésicos no Narcóticos, Náusea y Vómito Posoperatorios, Complicaciones Posoperatorias, Dolor Postoperatorio

## Abstract

Se realizó un metaanálisis para analizar el impacto de la anestesia sin opioides en las complicaciones postoperatorias de cirugía ginecológica (CG). Se seleccionaron 10 estudios con 852 mujeres sometidas a CG al inicio del estudio. Se utilizaron odds ratios (OR), diferencias de medias (DM) e intervalos de confianza del 95% (IC 95%) para analizar los efectos de la O-FA en la CG, en comparación con el tratamiento control (anestésico a base de opioides), mediante métodos dicotómicos o continuos, utilizando un modelo de efectos fijos o aleatorios. Las mujeres sometidas a GS, O-FA se asociaron con náuseas y vómitos posoperatorios significativamente menores (OR, 0,33; IC del 95 %, 0,24 – 0,46, p < 0,001), menor antiemético de rescate (OR, 0,32; IC del 95%, 0,13 – −0,78, p = 0,01) y mayor Calidad de Recuperación-40 (MD, 4,69; IC del 95%, 2,48 – 6,90, p < 0,001) en comparación con el tratamiento de control. Sin embargo, no se observó diferencia significativa entre la extubación (DM: −0,61; IC del 95%, −1,45 – 0,22; p = 0,15), la puntuación de dolor posoperatorio (DM: 0,49; IC del 95 %, −0,64 – 1,62; p = 0,39) y el uso de analgésicos (OR: 1,77; IC del 95%, 0,57 – 5,50; p = 0,33) en mujeres con EG. En mujeres sometidas a EG, la O-FA resultó en una reducción significativa de náuseas y vómitos posoperatorios, una menor necesidad de antieméticos de rescate y una mayor Calidad de Recuperación-40 en comparación con la anestesia con opioides.

## INTRODUCCIÓN

Los opioides desempeñan un papel importante en la anestesia general, ya que proporcionan potentes efectos antinociceptivos y analgésicos intraoperatorios, al tiempo que ayudan a mantener estables los signos vitales durante las intervenciones quirúrgicas^1,2)^. Por lo tanto, el uso inadecuado de opioides puede provocar diversos efectos adversos, como náuseas y vómitos posoperatorios (Postoperative Nausea and Vomiting - P-ONV), depresión respiratoria, respuestas inflamatorias e hiperalgesia, lo que prolonga la duración de la hospitalización posoperatoria y eleva los gastos médicos^3)^. Además, el uso de opioides posoperatorios puede provocar adicción y abuso de opioides^4)^. La prevalencia de P-ONV en pacientes sometidas a cirugía ginecológica (CG) con administración de opioides oscila entre el 50% y el 80%, y estas pacientes suelen experimentar bradicardia e hipoxemia posoperatoria debido a los efectos residuales de los opioides^5,6)^. El creciente énfasis en una mayor recuperación después de la cirugía ha llevado a una mayor adopción de la Anestesia Sin Opioides (Opioid-Free Anesthetics - O-FA) en diversos procedimientos quirúrgicos^7)^. La O-FA es un enfoque de anestesia multimodal que combina diversos agentes farmacológicos, entre ellos sedantes, antagonistas del receptor N-metil-D-aspartato, anestésicos locales, medicamentos antiinflamatorios y agonistas del receptor α^2^, con el fin de optimizar la analgesia intraoperatoria y minimizar el uso de opioides durante la fase perioperatoria. Elkassabany y Mariano^8)^ describen la O-FA como una estrategia perioperatoria que se extiende desde el ingreso hasta el alta, centrada en técnicas no opioides para la anestesia y la analgesia, junto con opioides reservados únicamente para el dolor que no puede controlarse con técnicas alternativas. Forget^9)^ proponen que la O-FA representa una combinación de múltiples estrategias de ahorro de opioides para lograr evitar por completo su uso. Mulier y Dekock^10)^ distinguen la O-FA de la analgesia sin opioides, haciendo hincapié en que la O-FA no implica la administración de opioides antes o durante la cirugía hasta que el paciente recupera la conciencia. Puede mitigar el riesgo de los efectos secundarios prevalentes relacionados con los opioides, que comprenden la depresión respiratoria posoperatoria y la P-ONV, al tiempo que disminuye la probabilidad de que el paciente se vuelva dependiente y adicto a los opioides^11)^. La O-FA se ha implementado con éxito en cirugías bariátricas y torácicas con resultados óptimos^12,13)^.Este metaanálisis tuvo como objetivo comparar y resumir los resultados posoperatorios de P-ONV, uso de antieméticos, puntuaciones de dolor, consumo de analgésicos, tiempo de extubación y puntuaciones de Calidad de Recuperación (Quality of Recovery-40 QoR-40) entre la O-FA y la anestesia basada en opioides en la cirugía ginecológica (CG).

## OBJETIVOS

El objetivo del estudio fue analizar el metaanálisis para examinar el efecto de la O-FA en las complicaciones posoperatorias tras la CG.

## MÉTODOS

### Criterios de Elegibilidad

Proporcionar una visión general de los estudios que examinan el efecto de la O-FA en las complicaciones posoperatorias tras la CG^14)^.

### Fuentes de Información

La investigación completa se presenta en la Figura 1. Se incluyeron los estudios que cumplían los siguientes criterios de inclusión^15,16)^:

**Figura 1:** Diagrama de flujo del procedimiento de la investigación. Zhejiang, China.

1. Los estudios eran observacionales, prospectivos, retrospectivos o ensayos controlados aleatorios (ECA).
2. Las participantes eran mujeres que se sometieron a una exploración y a una CG.
3. El dolor posoperatorio se incluyó en el estudio.
4. El estudio comparó el efecto de la O-FA en las complicaciones posoperatorias tras una CG.

Se excluyeron los estudios en los que el resultado de los O-FA solo se comparaba con el control en la CG, que solo incluían el grupo de tratamiento de control o que carecían de comparaciones estadísticamente significativas^17,18)^.

### Estrategia de Búsqueda

Se aplicó la perspectiva PICOS para identificar el procedimiento del protocolo de búsqueda, que se caracterizó de la siguiente manera: la P «población», como las mujeres sometidas a CG; la O-FA fue la I «intervención», que consistió en anestesia sin opioides (O-FA); la C «comparación» fue la O-FA; la O «outcome - resultado» incluyó las complicaciones posoperatorias; y el S «Study design - diseño del estudio» no tuvo restricciones^19)^. Utilizando un conjunto de palabras clave y términos de búsqueda (Cuadro 1), realizamos una búsqueda exhaustiva en las bases de datos Cochrane Library, Google Scholar, Embase, PubMed y OVID hasta febrero de 2025^20,21)^. Para evitar incluir estudios que no fueran relevantes para el efecto de la O-FA en las complicaciones posoperatorias tras la CG, también eliminamos los registros duplicados. Los artículos restantes se recopilaron en una base de datos EndNote y se examinaron sus títulos y resúmenes para su evaluación^22,23)^.

**Cuadro 1:** Estrategia de búsqueda en bases de datos para la inclusión de estudios. Zhejiang, China.

Base de datos	Estrategia de búsqueda
**Google Scholar**	#1 “gynecological surgery” OR “opioid-free anesthetic” #2 “postoperative nausea and vomiting” OR “postoperative complication” OR “postoperative pain” #3 #1 AND #2
**Embase**	#1 ‘gynecological surgery’ /exp OR ‘opioid-free anesthetic’ #2 ‘postoperative nausea and vomiting’/exp OR ‘postoperative complication’/exp OR ‘postoperative pain’ #3 #1 AND #2
**Cochrane library**	#1 (gynecological surgery):ti,ab,kw OR (opioid-free anesthetic):ti,ab,kw (Word variations have been searched) #2 (postoperative nausea and vomiting):ti,ab,kw OR (postoperative complication):ti,ab,kw OR (postoperative pain):ti,ab,kw (Word variations have been searched) #3 #1 AND #2
**Pubmed**	#1 “gynecological surgery”[MeSH] OR “opioid-free anesthetic”[All Fields] #2 “postoperative nausea and vomiting”[MeSH Terms] OR “postoperative complication”[MeSH] OR “postoperative pain”[All Fields] #3 #1 AND #2
**OVID**	#1 “gynecological surgery”[All Fields] OR “opioid-free anesthetic” [All Fields] #2 “postoperative nausea and vomiting”[ All fields] OR “postoperative complication”[All Fields] OR “postoperative pain” [All Fields] #3 #1 AND #2

### Proceso de Selección

El proceso posterior se organizó y analizó de acuerdo con las directrices epidemiológicas y se examinó utilizando los métodos de metaanálisis^24,25)^.

### Proceso de Recolección de Datos

En este estudio, extraemos datos como el nombre del autor, los datos de la investigación, el año de la investigación, el país o la ubicación, el tipo de población, las categorías, las metodologías de estimación cuantitativa y cualitativa, las fuentes de datos, la estimación de las consecuencias, las fisonomías médicas y terapéuticas, y el análisis estadístico, que fueron algunos de los criterios utilizados para recolectar los datos^26)^.

### Elementos de Datos

Cuando un estudio informaba de valores diversos, los datos se extraían basándose en la evaluación del efecto de la O-FA en las complicaciones posoperatorias tras la CG.

### Evaluación del Riesgo de Sesgo de la Investigación

El riesgo de sesgo y la calidad metodológica de la investigación seleccionada fueron examinados de forma independiente por dos autores. La metodología de cada estudio se revisó objetivamente para garantizar su precisión y fiabilidad.

### Medidas de Efecto

El análisis de sensibilidad se limitó a los estudios que evaluaban el efecto de la O-FA en las complicaciones posoperatorias tras la CG. Se llevó a cabo un análisis de subclases para comparar la relación entre la O-FA y el control (anestésico opioide) en diversas variables de los pacientes en la sensibilidad de los pacientes con CG.

### Métodos de Síntesis

Se calcularon el IC del 95%, la diferencia de medias (DM) y la odds ratio (OR) para el enfoque de datos continuos y dicotómicos utilizando modelos de efectos fijos o aleatorios. La heterogeneidad se calculó mediante la estadística I^2^, con un valor del 0% al 100%. Se observó heterogeneidad nula, baja, moderada y alta en el 0%, el 25%, el 50% y el 75% de los datos, respectivamente^27)^. Se realizaron análisis adicionales de los estudios con características similares para garantizar la selección del modelo adecuado. Si I^2^era inferior al 50%, se eligió el efecto fijo; en caso contrario, se aplicó el efecto aleatorio^27)^. Mediante la división de la estimación inicial en los grupos de consecuencias previamente designados, se llevó a cabo un análisis de subclases. El análisis utilizó un valor p inferior a 0,05 para determinar si los cambios entre las subcategorías eran estadísticamente significativos.

### Evaluación del Sesgo de Notificación

Se utilizaron la prueba de regresión de Egger y los gráficos de embudo, que representan el logaritmo de los OR o DM frente a sus errores estándar, como técnicas cuantitativas y cualitativas para evaluar el sesgo de publicación. Un p ≥ 0,05 indicaba la presencia de sesgo de investigación^28)^.

### Evaluación de la Certeza

Se aplicaron pruebas bilaterales para todos los valores p. Se utilizó Reviewer Manager Versión 5.3 (The Nordic Cochrane Centre, the Cochrane Collaboration, Copenhague, Dinamarca) para los análisis estadísticos y las representaciones gráficas.

## RESULTADOS

Se seleccionaron para el estudio un total de 10 publicaciones que cumplían los criterios de inclusión y que se publicaron entre 1999 y 2025, de entre 867 estudios relacionados^(29–38)^. Todas las investigaciones seleccionadas fueron ensayos controlados aleatorios. Los resultados de estos estudios se muestran en la Tabla 1. En total, se incluyeron 852 mujeres con CG al inicio de las investigaciones. El tamaño de la muestra de los estudios seleccionados osciló entre 40 y 152 pacientes. Como se ilustra en las Figuras 2–4, en las mujeres con CG, el O-FA tuvo un P-ONV significativamente menor (OR, 0,33; IC del 95%, 0,24 – 0,46, p < 0,001) con baja heterogeneidad (I^2^= 32%), menor antiemético de rescate (OR, 0,32; IC del 95%, 0,13 – 0,78, p = 0,01) con heterogeneidad moderada (I^2^= 63%) y mayor QoR-40 (DM, 4,69; IC del 95%, 2,48 – 6,90, p < 0,001) con baja heterogeneidad (I^2^= 36%) en comparación con el tratamiento de control (anestésico opioide).

**Tabla 1:** Calidades de los estudios seleccionados para el metaanálisis Zhejiang, China.

Estudio	País	Total	Anestesia sin opioides	Control
Callesen, 1999^29)^	Dinamarca	40	20	20
Hakim, 2019^30)^	Egipto	80	40	40
Massoth, 2021^31)^	Alemania	152	76	76
Choi, 2022^32)^	Corea	75	37	38
Cha, 2023^33)^	China	90	45	45
Chen, 2023^34)^	China	77	39	38
Koo, 2023^35)^	Corea	96	48	48
Hu, 2024^36)^	China	72	36	36
Nam, 2024^37)^	Corea	120	60	60
Kalagara, 2025^38)^	India	50	25	25
	Total	852	426	426

**Figura 2:** Forest plot de la influencia de la anestesia sin opioides en comparación con el tratamiento de control sobre las náuseas y los vómitos posoperatorios en participantes sometidas a cirugía ginecológica. Zhejiang, China.

**Figura 3:** Forest plot de la influencia de la anestesia sin opioides en comparación con el tratamiento de control en el uso de antieméticos de rescate en participantes con cirugía ginecológica en Zhejiang, China.

**Figura 4:** Forest plot de la influencia de la anestesia sin opioides en comparación con el tratamiento de control en el QoR-40 en participantes con cirugía ginecológica, Zhejiang, China

Sin embargo, no se observaron diferencias significativas entre el O-FA y el tratamiento de control en el tiempo de extubación (DM, -0,61; IC del 95%, −1,45 – 0,22, p = 0,15) con heterogeneidad moderada (I^2^ = 68%), la puntuación del dolor posoperatorio (DM, 0,49; IC del 95%, −0,64 – 1,62, p = 0,39) con alta heterogeneidad (I^2^ = 86%) y el uso de analgésicos (OR, 1,77; IC del 95%, 0,57 – 5,50, p = 0,33) con alta heterogeneidad (I^2^ = 79%) en mujeres con GS, como se muestra en las Figuras 5–7.

**Figura 5:** Forest plot de la influencia de la anestesia sin opioides en comparación con el tratamiento de control en el tiempo de extubación en participantes con cirugía ginecológica en Zhejiang, China.

**Figura 6:** Forest plot de la influencia de la anestesia sin opioides en comparación con el tratamiento de control en la puntuación del dolor posoperatorio en participantes con cirugía ginecológica en Zhejiang, China.

**Figura 7:** Forest plot de la anestesia sin opioides en comparación con el tratamiento de control en el uso de analgésicos en participantes con cirugía ginecológica en Zhejiang, China.

El uso de modelos estratificados para evaluar los efectos de factores específicos no fue viable debido a la insuficiencia de datos, como el género, el origen étnico y la edad, en las consecuencias de la comparación. No se encontraron pruebas de sesgo de publicación mediante la aclaración visual del gráfico de embudo y la prueba de regresión cuantitativa de Egger (p = 0,88, 0,89, 0,89, 0,91, 0,86 y 0,91, respectivamente). Sin embargo, la mayoría de los ECA incluidos mostraron una calidad metodológica subóptima, aunque no se observaron pruebas de sesgo de selección en los informes.

## DISCUSIÓN

Se necesitan más investigaciones para validar estos hallazgos, ya que varias comparaciones se basaron en un número limitado de estudios con muestras de tamaño reducido, lo que puede influir en la fiabilidad de los resultados.

El metaanálisis incluyó a 852 mujeres con CG^(29–38)^. En las mujeres, el O-FA se relacionó con una P-ONV significativamente menor, una menor necesidad de antieméticos de rescate y puntuaciones QoR-40 más altas en comparación con el tratamiento de control (anestésico opioide). Sin embargo, no se observaron diferencias significativas en el tiempo hasta la extubación, la puntuación del dolor postoperatorio o el uso de analgésicos en las mujeres con CG. Se necesitan más investigaciones para confirmar estos hallazgos y se debe tener precaución al interpretar los resultados, ya que varias comparaciones se basaron en un número limitado de estudios, muchos de los cuales tenían muestras de tamaño reducido. Esto puede afectar a la fiabilidad y la significación generales de las evaluaciones revisadas.

A pesar de las numerosas investigaciones previas que han examinado los resultados postoperatorios de la anestesia O-FA y la anestesia basada en opioides, sigue habiendo una falta de revisiones sistemáticas y metaanálisis centrados en un procedimiento quirúrgico específico. Dos metaanálisis informaron de que la O-FA reducía el dolor postoperatorio en los pacientes^39,40)^, y uno indicó un tiempo de extubación más corto^40)^, lo que contrasta con nuestros hallazgos. La O-FA sustituye los opioides por medicamentos alternativos o métodos anestésicos durante la anestesia, lo que reduce el uso perioperatorio de opioides y puede disminuir la aparición de P-ONV, en consonancia con los hallazgos de este metaanálisis. Callesen et al. emplearon anestesia espinal-epidural combinada en el grupo de O-FA^29)^, y la anestesia regional puede presentar un riesgo distinto de P-ONV en comparación con la anestesia general, lo que influye en la precisión de los datos^41)^. La P-ONV sigue siendo un reto clínico importante. El sexo femenino es un factor de riesgo intencional para la P-ONV, con una prevalencia notablemente mayor observada en las GS en comparación con otros tipos de cirugías^42)^. Apfel et al. informaron de que la incidencia de P-ONV puede alcanzar hasta el 80% en las GS sometidas a anestesia basada en opioides^5,43)^. Aunque los opioides se utilizan comúnmente como analgésicos perioperatorios debido a sus potentes efectos analgésicos, también están relacionados con efectos secundarios, como náuseas y vómitos^44)^. Desde el punto de vista mecánico, los opioides pueden inducir la P-ONV mediante la estimulación directa de los receptores de la zona desencadenante de los quimiorreceptores del tronco encefálico^45)^.

Además, hay pruebas sustanciales que demuestran que tanto la prevalencia como la gravedad de la P-ONV dependen de la dosis de opioides perioperatorios^46)^. Entre las investigaciones incluidas, cuatro incorporaron dexmedetomidina como parte de la O-FA. Investigaciones previas indican que la dexmedetomidina, un agonista del receptor α^2^, puede disminuir la incidencia de la P-ONV al modular la liberación de 5-hidroxitriptamina y dopamina^47)^. A pesar de las diferentes combinaciones de agentes anestésicos en los distintos estudios, nuestros hallazgos sugieren que la O-FA proporciona un beneficio en la reducción de la P-ONV durante las intervenciones ginecológicas. El efecto de la O-FA sobre el dolor postoperatorio sigue sin ser concluyente; este metaanálisis no encontró diferencias estadísticamente significativas en las puntuaciones de dolor postoperatorio o en las necesidades de analgésicos entre los grupos de O-FA y los grupos de control. Estos hallazgos respaldan el uso de la analgesia multimodal para reducir el consumo de opioides y lograr un alivio adecuado del dolor. Sin embargo, las variaciones en los protocolos analgésicos posoperatorios y los regímenes de analgesia multimodal entre los distintos estudios limitan la fiabilidad de esta conclusión, lo que pone de relieve la necesidad de realizar más ensayos clínicos para definir mejor la relación entre la O-FA y el dolor posoperatorio. Además, no se observaron diferencias significativas en el tiempo de extubación entre los dos grupos. Aunque en general se espera que el uso de opioides prolongue la extubación debido al aumento de la sedación, este hallazgo contradice las hipótesis convencionales.

No se observaron diferencias en el tiempo de extubación entre los dos grupos. Aunque normalmente se espera que el uso de opioides aumente la sedación y prolongue el tiempo de extubación, este hallazgo contradice las hipótesis convencionales y puede atribuirse al tamaño limitado de la muestra, lo que reduce su fiabilidad. El metaanálisis también mostró que la O-DA mejoraba la calidad de la recuperación. La investigación incluida evaluó la calidad de la recuperación. La investigación incluida examinó la recuperación utilizando la escala QoR-40, en la que el P-ONV es un elemento significativo de la escala QoR-40; por lo tanto, la reducción de la incidencia de P-ONV puede mejorar la calidad de la recuperación posoperatoria. No obstante, el número limitado de estudios incluidos y la variabilidad significativa restringen la confianza en los resultados secundarios.

### Limitaciones

La principal limitación de esta revisión es que la mayoría de las investigaciones incluidas carecían de datos sobre la estabilidad lumbar, el grado de deslizamiento y el síntoma principal de los pacientes, lo que impidió realizar un análisis estratificado más detallado. Además, no se disponía de información suficiente para examinar el efecto potencial de la edad, el sexo o la raza en los resultados. Esta restricción nos impidió realizar un análisis estratificado adicional. Además, carecíamos de datos suficientes para evaluar la influencia potencial de la edad, el sexo y la raza en los resultados. La inclusión de datos incompletos o inexactos de investigaciones anteriores puede haber introducido un sesgo. Sin embargo, es probable que las investigaciones no comunicadas y la información que faltaba, como la edad, el sexo, la raza y la nutrición de los participantes, estuvieran sesgadas. Los datos incompletos y las investigaciones no comunicadas pueden dar lugar a valores que están inadvertidamente sesgados.

## CONCLUSIONES

En las mujeres sometidas a cirugía ginecológica, el O-FA se asoció con una P-ONV significativamente menor, una menor necesidad de antieméticos de rescate y una puntuación QoR-40 más alta en comparación con el tratamiento de control (anestésico opioide). Sin embargo, no se observaron diferencias significativas en el tiempo de extubación, la puntuación del dolor posoperatorio y el uso de analgésicos. Se necesitan más investigaciones para validar estos hallazgos, ya que varias comparaciones se basaron en un número limitado de investigaciones, algunas de ellas con muestras de tamaño reducido, lo que puede afectar a la fiabilidad y la significación de los resultados.

## Data Availability

Los conjuntos de datos analizados durante el presente estudio están disponibles previa solicitud razonable al autor correspondiente.
